# Hypersensitivity of BRCA2 deficient cells to rosemary extract explained by weak PARP inhibitory activity

**DOI:** 10.1038/s41598-017-16795-3

**Published:** 2017-12-01

**Authors:** Cathy Su, Jeffrey P. Gius, Julia Van Steenberg, Alexis H. Haskins, Kazuki Heishima, Chisato Omata, Masahiro Iwayama, Mami Murakami, Takashi Mori, Kohji Maruo, Takamitsu A. Kato

**Affiliations:** 1 Department of Environmental and Radiological Health Sciences, Colorado State University, Fort Collins, CO 80523 USA; 2 Faculty of Applied Biological Sciences, United Department of Veterinary Medicine, Gifu University, 1–1, Yanagido Gifu, 501–1193 Japan

## Abstract

Rosemary extract is used in food additives and traditional medicine and has been observed to contain anti-tumor activity. In this study, rosemary extract is hypothesized to induce synthetic lethality in BRCA2 deficient cells by PARP inhibition. Chinese hamster lung V79 cells and its mutant cell lines, V-C8 (BRCA2 deficient) and V-C8 with BRCA2 gene correction were used. Rosemary extract and its major constituent chemicals were tested for their cytotoxicity by colony formation assay in cells of different BRCA2 status. The latter chemicals were tested for inhibitory effect of poly (ADP-ribose) polymerase (PARP) activity *in vitro* and *in vivo*. Rosemary has shown selective cytotoxicity against V-C8 cells (IC_50_ 17 µg/ml) compared to V79 cells (IC_50_ 26 µg/ml). Among tested chemicals, gallic acid and carnosic acid showed selective cytotoxicity to V-C8 cells along with PARP inhibitory effects. Carnosol showed comparative PARP inhibitory effects at 100 µM compared to carnosic acid and gallic acid, but the selective cytotoxicity was not observed. In conclusion, we predict that within rosemary extract two specific constituent components; gallic acid and carnosic acid were the cause for the synthetic lethality.

## Introduction

Rosemary, *Rosmarinus officinalis*, is a common evergreen herb frequently used in cooking and traditional medicine^1^. Rosemary extract contains essential oils, terpenoids, flavonoids, and alkaloids^1^ including rosmarinic acid, carnosic acid, carnosol, tannic acid, and gallic acid which have been the focus of many experiments centered around the cytotoxicity, anti-inflammatory, and genotoxicity of the purified components of rosemary^2–4^. Recently, studies have theorized that rosemary extract can provide significant antiproliferative effects on many different cancer cell lines^5,6^. Specifically, it has been proposed that two compounds, carnosic acid and rosmarinic acid, are the cause for the observed anti-tumor affect in various human cancer cell lines including lung, prostate, liver, and breast cancers^7^. Because of this, cancer research centered on the effects of rosemary extract is very promising.

Breast cancer is one of the most common cancers in women, and BRCA2 deficiency is a clear genetic mutation that increases the risk of hereditary breast cancer. Although genetic factors are not the primary cause of breast cancer, only accounting for approximately 5–10% of all cases, this is a factor that cannot be controlled by the patient or managed through a physician with the exception of a mastectomy^8^. BRCA2 is a human tumor suppressor gene involved in homologous recombination (HR) repair and is observed to be commonly mutated in breast tumors^9^. Women with the heterozygous mutation suffer from a large increased risk of developing breast cancer in their lifetime. They also have an increased risk of developing other cancers, including ovarian cancers^10^. However, recent studies suggest that the inhibition of an enzyme associated with single strand break repair, poly (ADP ribose) polymerase (PARP), in BRCA1/2 homozygous mutated cells results in selective cell killing^11,12^. This synthetic lethality occurs in BRCA1/2 homozygous mutated cancer cells but not BRCA1/2 heterozygous normal tissues, making this a favorable target for potential cancer therapy or preventative treatments. In our previous studies, it was observed that natural flavonoid chemicals, similar to the 24 identified flavonoids found in rosemary^13^, often have PARP inhibitory effects^14,15^. We hypothesized that rosemary extract may contain chemicals that have PARP inhibitory activity that would result in selective killing of BRCA1/2 deficiency cells. Expanding this research might be a viable option in developing new types of therapeutic treatments. By combining current chemotherapy treatment options with proposed PARP inhibitory treatments, the sensitivity of HR deficient cancer types may increase; thus, both decreasing unwanted side effects and increasing projected patient survival rates^16,17^.

We used BRCA2 deficient cells commonly found in familial breast tumors. The potential for rosemary extract to inhibit PARP was investigated as one of the possible primary mechanisms of action. We suggest that this led to the selective killing of the BRCA2 deficient cells.

## Results

### BRCA2 selective cytotoxic effect of rosemary extract

**Figure 1:**
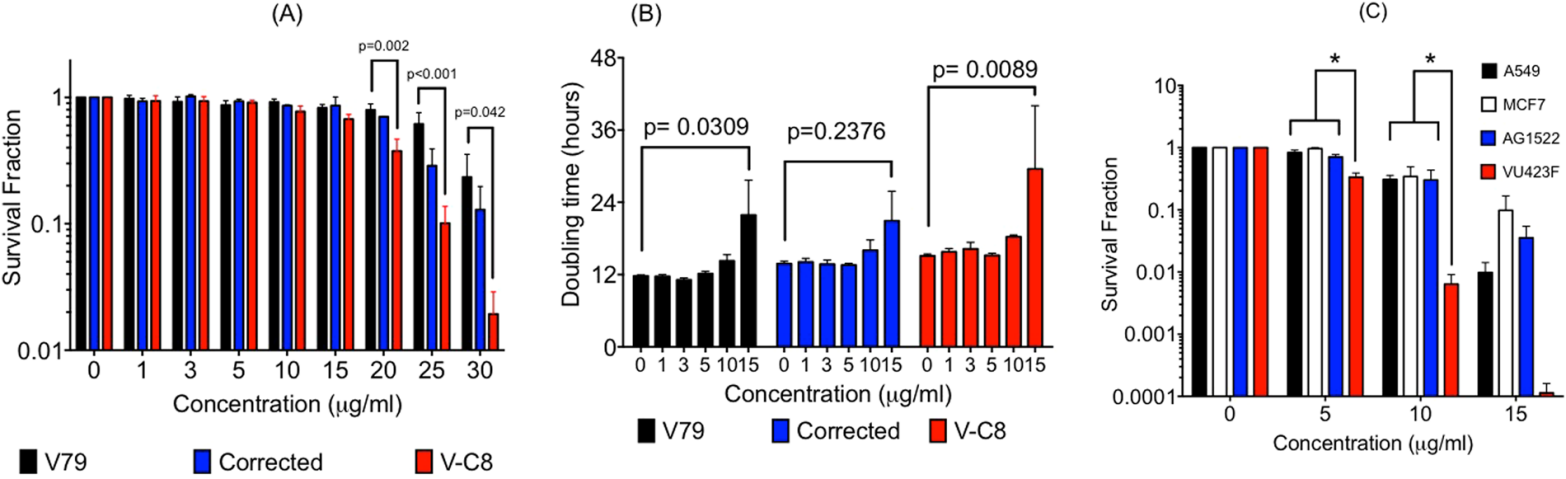
Cytotoxicity to rosemary extract. (**A**) Cell survival fraction obtained by colony formation assay for hamster cells. Black bars indicate V79, blue bars indicate gene corrected V-C8, and red bars indicate V-C8 cells. (**B**) Elongation of cell doubling time with high concentrations of rosemary extract exposure. (**C**) Cell survival fraction obtained by colony formation assay for human cells. Black bars indicate A549, white bars indicate MCF7, blue bars indicate AG1522, and red bars indicate VU423F BRCA2 deficient cells. At least three independent experiments were carried out. Error bars indicate standard error of the mean. P-values represent two-way ANOVA results. *symbols indicate statistical significance (P < 0.05).

Chinese hamster lung V79 cells and its mutant cell lines, V-C8 (BRCA2 deficient) and V-C8 with BRCA2 gene correction, were used for colony formation assay to determine the effect of rosemary extract (Fig. 1A). IC_50_ of clonogenicity for V-C8 cells under treatment of rosemary extract was observed to be 17.8 µg/ml, while IC_50_ of clonogenicity for V79 cells and gene corrected cells were 26.5 and 22.7 µg/ml, respectively. Rosemary extract showed a selective cytotoxicity to V-C8 cells. Statistically significant differences for survival rates were observed at the concentration above 20 µg/ml between V79 and V-C8 cells (Fig. 1A). Cytotoxicity was also assessed through cell doubling time (Fig. 1B). Cell doubling time of V79 was measured to be 12 hours without treatment. Rosemary extract in the culture media showed the ability to postpone cell growth in all cell lines above 10 µg/ml. Cell doubling time of V79, originally 12 hours increased to 22 hours (P = 0.0309), and V-C8 changed from 15 hours to 30 hours (P = 0.0089). However, gene corrected cells yielded a smaller change of 14 hours to 21 hours (P = 0.23) following 15 µg/ml rosemary extract treatment. Therefore, the differences in cell doubling time were marginal and were not dependent on BRCA2 deficiency.

Additional colony formation assay was conducted to confirm cytotoxicity dependence on BRCA2 deficiency. In comparison to both normal and cancerous cell lines, Fig. 1C shows that the cytotoxicity of rosemary extract was more severe in VU423F, a Fanconi anemia cell line with BRCA2 mutations. At 5 µg/ml, selective cytotoxicity to VU423F cells had P-values of 0.0012 for A549, 0.0001 for MCF7, and 0.0026 for AG1522. At 10 µg/ml, P-values were 0.007 for A549, 0.0266 for MCF7, and 0.0199 for AG1522. At 15 µg/ml, BRCA2 normal cells showed cytotoxicity and no significant differences were observed in comparison to VU423F.

### *In vitro* PARP inhibitory effect and DNA damage formation by rosemary extract

**Figure 2:**
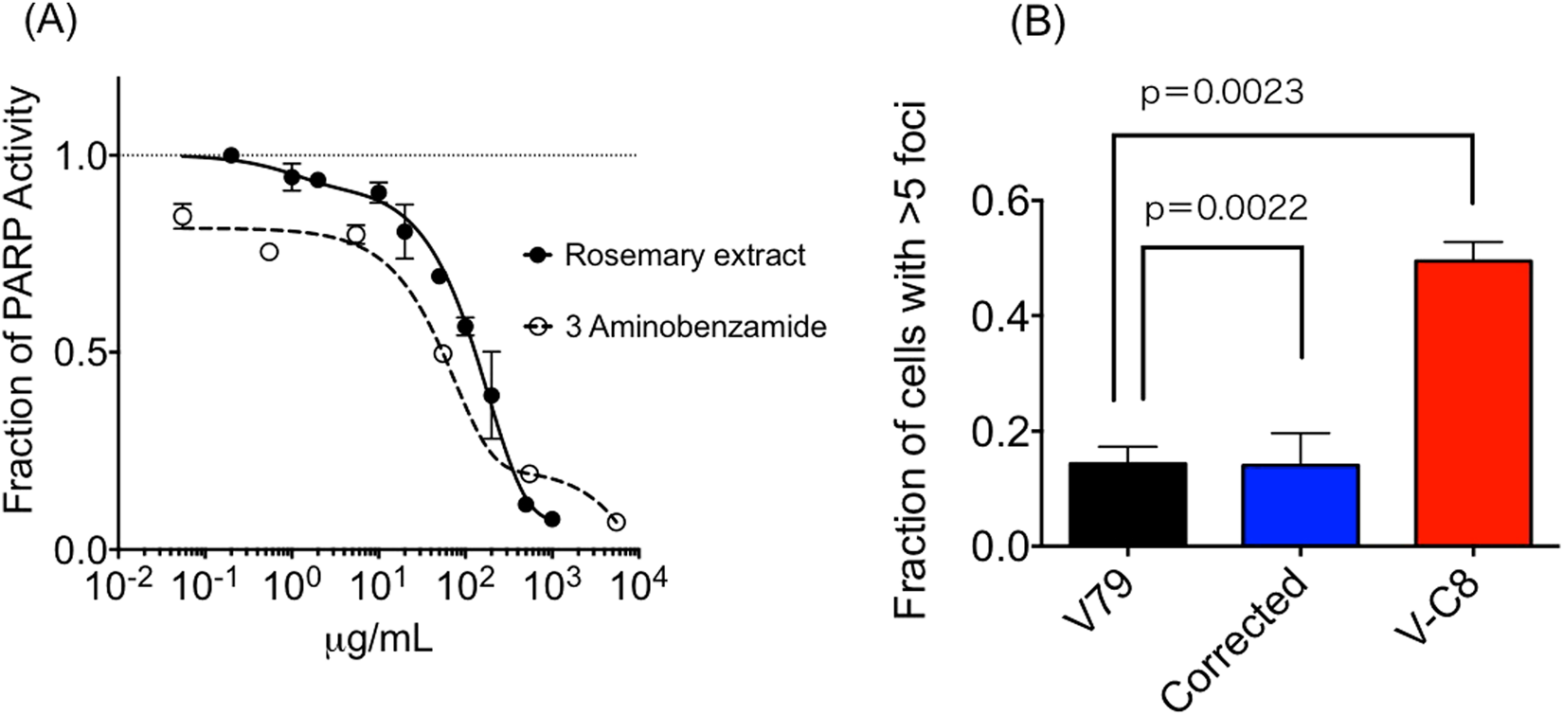
*In vitro* PARP inhibitory effect and DNA damage formation by rosemary extract. (**A**) PARP activity in the presence of rosemary extract or 3 aminobenzamide. (**B**) Massive DNA double strand break formation after overnight rosemary extract treatment. At least three independent experiments were carried out. Error bars indicate standard error of the mean. P-values smaller than 0.05 were considered to be statistical significant.

Rosemary extract was tested for *in vitro* PARP inhibitory capability. IC_50_ value of PARP activity inhibition was approximately 130 µg/ml. Positive control 3-aminobenzamide showed stronger PARP inhibitory capacity with IC_50_ value of 55 µg/ml (Fig. 2A). Rosemary extract was observed as weaker but comparative PARP inhibitor when compared to 3-aminobenzamide.

Rosemary extract was tested for BRCA2 dependent DNA damage formation (Fig. 2B). 20 µg/ml of rosemary extract treatment overnight induced massive DNA double strand break formation, categorized as more than 5 gamma-H2AX foci per cell in V-C8 cells (50%) but less frequently observed in V79 and gene corrected cells (15%).

### *In vitro* PARP inhibitory effects of the major rosemary extract compounds

**Figure 3:**
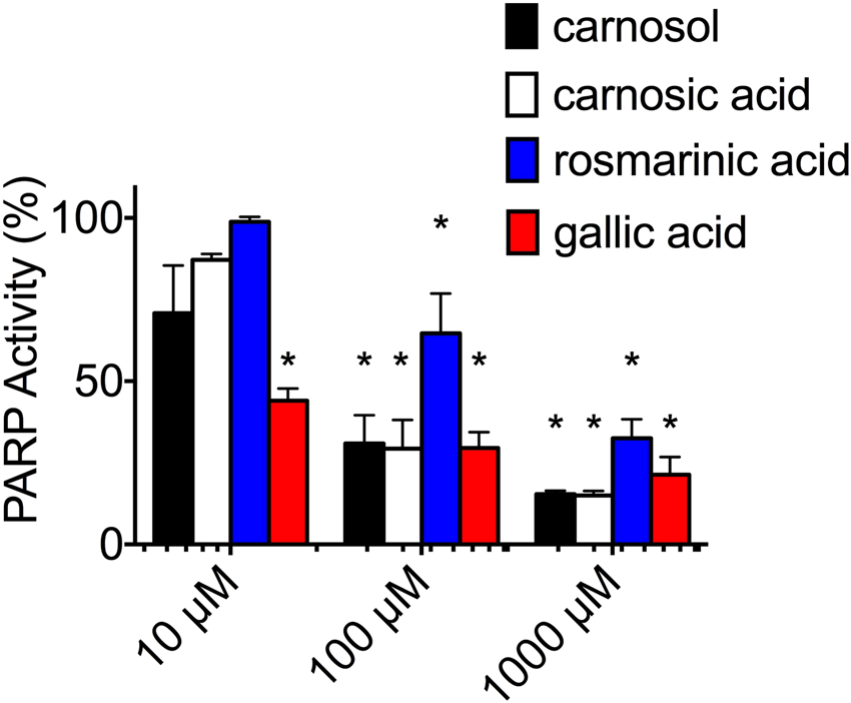
*In vitro* PARP inhibitory effect of carnosol, carnosic acid, rosmarinic acid, and gallic acid. At least three independent experiments were carried out. Error bars indicate standard error of the mean. *symbol indicates statistical significant reduction compared to control (P < 0.05).

10, 100 and 1000 µM solutions of carnosic acid, carnosol, rosmarinic acid, and gallic acid were tested for PARP inhibitory effect *in vitro* (Fig. 3). Dose dependent PARP inhibitory effect was observed. A 100 µM concentration of carnosol, carnosic acid, and gallic acid showed similar PARP inhibitory effects, which inhibited 70% of PAR formation. However, a 100 µM concentration of rosmarinic acid did not show strong inhibitory effects (20% inhibition) compared to the other three tested compounds. IC_50_ values were calculated as 6, 35, 50, and 200 µM for gallic acid, carnosol, carnosic acid, and rosmarinic acid, respectively.

### *In vivo* PARP inhibitory effects of the rosemary extract and its major compounds

**Figure 4:**
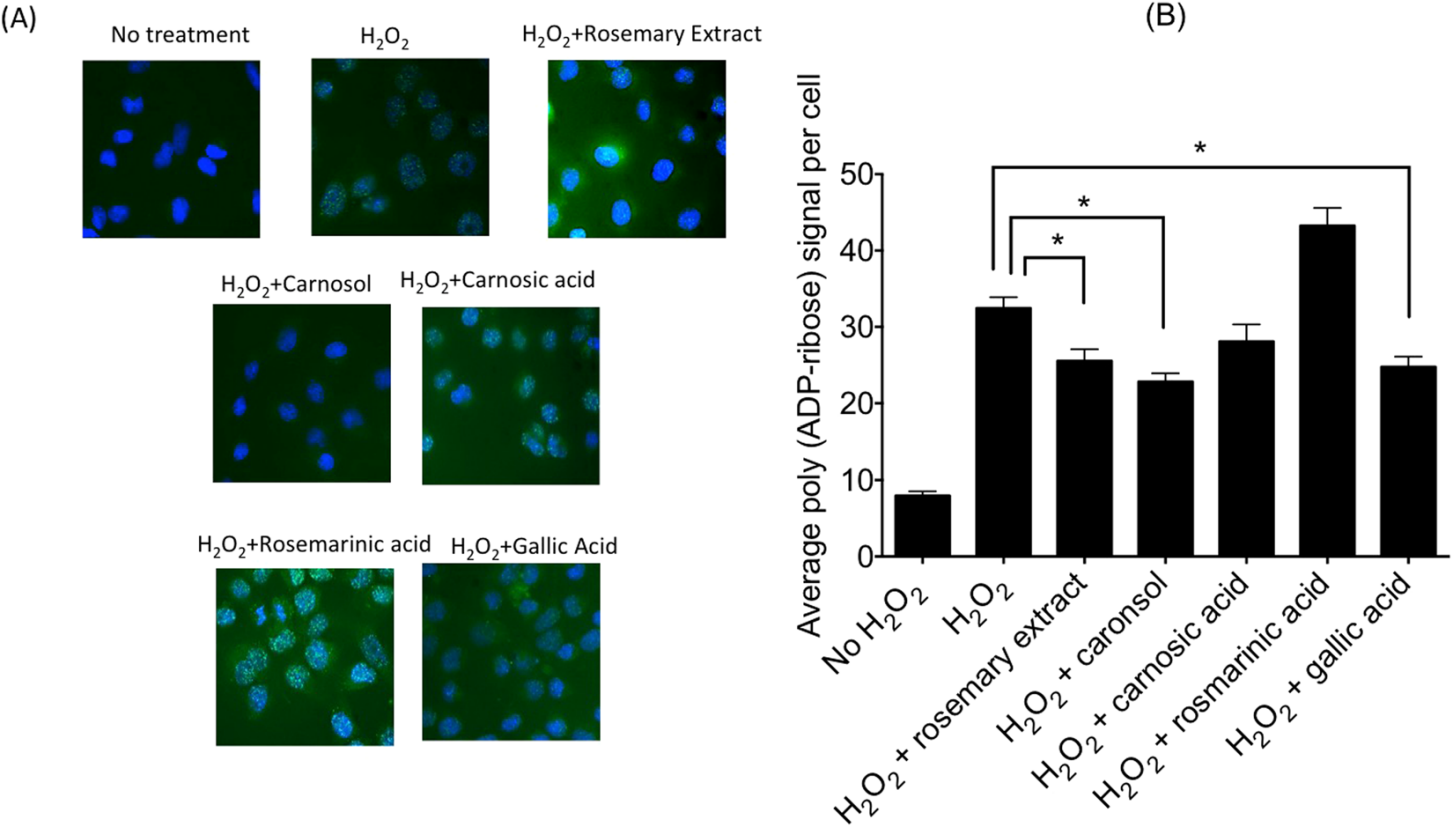
*In vivo* PARP inhibitory effect by rosemary extract and its major compounds. (**A**) Representative images of H_2_O_2_ induced poly (ADP-ribose) formation as green signals. Blue signals are nuclei. (**B**) Quantitative analysis of poly (ADP-ribose) formation by green pixel analysis. Average green pixels per cell (arbitrary unit). At least three independent experiments were carried out. Error bars indicate standard error of the mean. *symbols indicate statistical significance (P < 0.05).

Prior to H_2_O_2_ treatment, 10 µg/ml of rosemary extract and 10 µM solutions of carnosic acid, carnosol, rosmarinic acid, and gallic acid were added to media and their PARP inhibitory effects were assessed by measuring poly (ADP-ribose) formation in cells (Fig. 4A). Rosemary extract (P = 0.0173), carnosol (P = 0.0007), and gallic acid (P = 0.0072) showed statistically significant reduction of poly (ADP-ribose) formation in the tested condition. Carnosic acid showed a reduction trend (78% of H_2_O_2_ control) but displayed no significant signal reduction (P = 0.3382). Observation of rosmarinic acid showed induction rather than noticeable reduction of fluorescence signals indicating poly (ADP-ribose) polymerization (Fig. 4B).

### BRCA2 selective cytotoxicity in the major compounds of rosemary extract

**Figure 5:**
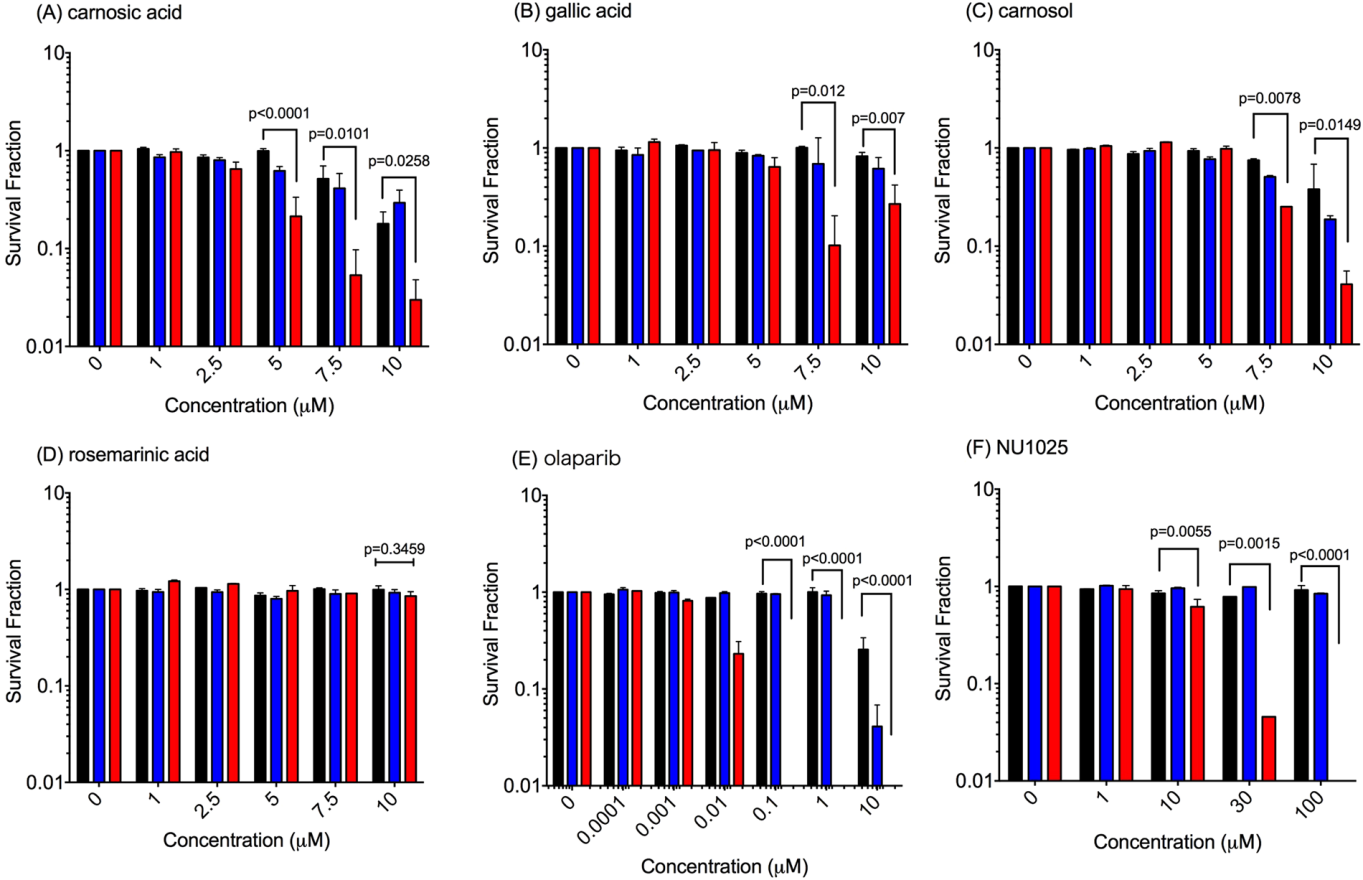
Cell survival curves against chemicals. (**A**) Carnosic acid (**B**) Gallic acid (**C**) Carnosol (**D**) Rosmarinic acid (**E**) Olaparib, (**F**) NU1025. Black bars indicate V79, blue bars indicate gene corrected V-C8, and red bars indicate V-C8 cells. At least three independent experiments were carried out. Error bars indicate standard error of the mean. P-values represent two-way ANOVA analysis between V79 and V-C8. P-values smaller than 0.05 were considered to be statistical significant.

To identify which compounds caused the observed selective cytotoxicity to BRCA2 deficient cells by rosemary extract treatment, carnosic acid, carnosol, rosmarinic acid, and gallic acid were investigated (Fig. 5A–D). These four chemicals were tested due to their ability to inhibit PARP. Among the four tested chemicals carnosol and gallic acid showed selective cytotoxicity to V-C8 cells with treatment concentrations greater than 7.5 µM. The IC_50_ of cell survival against carnosol and gallic acid in BRCA2 deficient cells was measured to be approximately 6 µM. Carnosic acid showed significant cytotoxic effects above the concentration of 5 µM. Cell survival against carnosic acid in BRCA2 deficient cells had a IC_50_ value of 4 µM. However, rosmarinic acid failed to show cytotoxicity effects in V79, V-C8, and gene corrected cells for all tested concentrations up to 10 µM.

Olaparib and NU1025, known selective PARP inhibitors, were used as positive controls (Fig. 5E–F). Olaparib and NU1025 showed severe selective cytotoxicity to BRCA2 deficient cells. In olaparib treated V79 cells, IC_50_ value of cell survival was measured at 10 µM and IC_50_ for cell survival in V-C8 cells was less than 0.01 µM. NU1025 showed selective cytotoxicity to BRCA2 deficient cells above 10 µM. It was observed that BRCA2 deficient cells required higher concentrations of NU1025 to display selective cytotoxicity compared to carnosic acid.

#### Assessment of contribution from major compounds

PARP inhibitory effect and cytotoxicity of rosemary extract were assessed by IC_50_ to identify the contribution of major compounds. Among four tested chemicals, two major compounds, carnosol and carnosic acid consists approximately 13.9% and 9.5% of rosemary extract. If these chemicals are major reasons to cause cytotoxicity and PARP inhibitory effect, they should be 10 times effective per concentration than rosemary extract. IC_50_ of *in vitro* PARP inhibitory effect was 130 µg/ml for rosemary extract, 34 µM (11 µg/ml) for carnosol and 50 µM (15 µg/ml) for carnosic acid. Therefore, carnosol and carnosic acid are approximately 10 times stronger PARP inhibitors compared to rosemary extract. Major part of PARP inhibitory effect in rosemary extract can be explained by carnosol and carnosic acid. In contrast, the IC_50_ of V-C8 cytotoxicity was 17.8 µg/ml for rosemary extract (Fig. 1A), 4 µ M (1.3 µg/ml) for carnosol, 5 µ M (1.6 µg/ml) for carnosic acid. V-C8 cells were 10 times sensitive to carnosol and carnosic acid than rosemary extract. Therefore, carnosol and carnosic acid can account major part of cytotoxicity in rosemary extract to V-C8 cells.

## Discussion

In this study, we showed rosemary extract can cause synthetic lethality in BRCA2 deficient cells through PARP inhibition (Figs. 1,2). In treated cells, cell doubling time is extended and rosemary extract may interfere with cell cycle progression and postpone cell growth. This result is consistent with previous studies, which have shown rosemary extract induced perturbation of cell cycle progression in human ovarian cancer cells^6^. Although growth delay was not BRCA2 deficiency dependent, clonogenic assay showed stronger cytotoxicity was induced by rosemary extract in BRCA2 deficient cells. However, due to the relatively weak PARP inhibitory effect, the ratio of IC_50_ for rosemary extract induced cytotoxicity between wild type BRCA2 cells and BRCA2 deficient cells was not as large as known selective PARP inhibitors, such as olaparib (Figs 1A and 5E)^18^. This ratio was relatively small and comparative to the natural flavonoids with PARP inhibitory effects^14^. Human cell line experiment data added extra support for BRCA2 deficiency dependent cytotoxicity by rosemary extract (Fig. 1C).

Rosemary extract contains many chemicals and several major chemical compounds including, carnosic acid, gallic acid, carnosol, and rosmarinic acid. These four chemicals were used in this study to further identify active PARP inhibitors. All tested chemicals showed *in vitro* PARP inhibitory effects. Carnosol, carnosic acid, and gallic acid showed *in vivo* PARP inhibitory effects; however, only carnosic acid and gallic acid showed significant cell killing in BRCA2 deficient cells (Fig. 5A–B). Previous studies have observed rosemary extract to reduce proliferation through Akt and other downstream pathways which can lead to apoptosis of the cell^19,20^. Our findings added rosemary extract for a novel cytotoxicity pathway by PARP inhibition and synthetic lethality.

Individual chemicals in rosemary extract have also been previously studied. Especially, the ability of carnosic acid and gallic acid to downregulate the Akt pathway resulting in apoptosis^21–23^. These chemicals were identified in our experimentation to contain strong PARP inhibitory effects and displayed synthetic lethality in BRCA2 deficient cells. Conversely, rosmarinic acid did not show synthetic lethality in BRCA2 deficient cells and had minimum inhibitory activity against PARP. On the other hand, carnosol had similar PARP inhibitory capacity, but showed no synthetic lethality in BRCA2 deficient cells. This observation can likely be explained by other dominant cytotoxic pathways such as antagonistic binding. Carnosol is a receptor antagonist which binds in the ligand binding domain of the androgen receptor and estrogen receptor-alpha^24^. The IC_50_ of cellular growth was reported as 25.6 µM in the breast cancer cell line MCF-7^24^. Carnosol has also showed wild type p53 dependent anti-proliferation effect in glioblastoma cells^25^. Carnosol induces apoptosis through down regulation of Bcl-2^26^. It is also known that carnosol is one of the strongest active biomolecules in rosemary extract; therefore, we assumed that the PARP inhibition induced synthetic lethality effects were not visible because of death signals from other pathways. Furthermore, our results suggested that rosemary extract may have a different mechanism of cytotoxicity other than PARP inhibition.

The bioavailablity of rosemary extract and important chemicals in rosemary have also been reported. Bioavailiability of drugs is a key factor to study before the standardization of preventative and chemotherapy treatments. Previous studies have shown that the bioavailablity of carnosic acid was 40% after oral injection in rats^27^, and 36–39% for gallic acid and is analog in the blood stream after oral ingestion in humans^28^. The bioavailability information for carnosol is unknown or limited. According to Vaquero’s pharmacokinetic parameters data, after oral administration of 100 mg of rosemary extract, the Tmax and Cmax of carnosol were 13.3 hours and 18.2 µM. However, the Tmax and Cmax values of carnosic acid, were 0.4 hours and 26.6 µM respectively^29^. The high bioavailability suggests that taking rosemary extract itself or purified carnosic acid or gallic acid as supplements can affect BRCA2 mutated cells in the body. It was clear that synthetic lethality induced by both rosemary extract and its chemicals were comparable to NU1025 but much lower than actual PARP inhibitor drugs, such as olaparib (Fig. 5). However, intake of rosemary extract and its chemicals can be easily increased in daily food additives and health supplements with relatively low cost. Rosemary extract is a known antioxidant and radical scavenger^3,30–33^: by combining these properties, it may be possible to show chemopreventive effects against BRCA2 deficient tumors in the individuals with BRCA2 heterozygous mutation by taking a low dose intake of rosemary chemicals daily.

In conclusion, this paper identified rosemary extract to contain PARP inhibitory activity and selective cell toxicity in BRCA2 deficient cells through synthetic lethality. This study also identified the main compounds in rosemary extract that act as PARP inhibitors which are proposed to be carnosic acid and possibly gallic acid. Further research should be done to investigate *in vivo* synthetic lethality induced chemoprevention and the PARP inhibitory mechanisms of these chemicals.

## Materials and Methods

### Cell culture

Chinese Hamster lung cell (V79), BRCA2 deficient mutant of this cell line (V-C8), and gene corrected mutant cells were used for all cell based assays, which were generously provided by Dr. Joel Bedford (Colorado State University, Fort Collins, CO)^34,35^. Normal human fibroblast AG1522, human breast carcinoma cell line MCF7, lung adenocarcinoma cell line A549, and BRCA2 defective VU423F cells were kindly supplied by Oregon Health and Science University Cell Repository (Portland, Oregon), and obtained from ATCC (Manassas, VA). Cells were maintained in culture in minimum essential medium alpha (MEMα, Gibco, Grand Island, NY), and supplemented with 10% heat inactivated fetal bovine serum (FBS, Sigma, St. Louis, MO) and 1% penicillin and streptomycin, and 0.1% fungizone solution (Gibco) in a humidified incubator at 37 °C and 5% CO_2_.

### Chemicals

Rosemary extract was provided by CCI Corporation (Gifu, Japan). The rosemary extract contained 13.8% of carnosol, 9.5% of carnosic acid, and 0.7% of rosmarinic acid. It was dissolved in DMSO to make a 50 mg/ml stock solution and stored at −20 °C. Carnosic acid, carnosol, gallic acid, and rosmarinic acid were purchased from Sigma (St Louis, MO). NU1025 and olaparib were obtained by Calbiochem (San Diego, CA).

### Doubling time assay

Ten thousand trypsinized cells were plated into 12-well cell culture plate. Cells were treated with various concentrations of rosemary extract after 2 hours of incubation. Cell numbers were counted by Coulter Counter Z1 (Beckman Coulter, Brea, CA) after 24, 48, 72, 96, and 120 hours of drug treatment. Cell doubling time were calculated through GraphPad Prism 7 (GraphPad software, Inc. La Jolla, CA).

### Cell survival assay

Exponentially growing cell cultures were trypsinized and approximately 300 cells were placed in cell culture dishes. Two hours after cells were seeded, the tested chemicals were added to the plates. Cells were incubated for 7–10 days to form colonies. Afterwards, cells were fixed with 100% ethanol and stained with 0.1% crystal violet, then manually counted. Colonies having more than 50 cells were considered to be a survivor.

### *In vitro* PARP activity Inhibition

HT Universal Colorimetric PARP Assay Kit (Trevigen, Gaithersburg, MD) was used to assess the capacity of rosemary extract as a PARP inhibitor. Solutions were prepared per directions provided with the kit from the manufacturer and 3-Aminobenzamide(3-AB) was used as a known PARP inhibitor. The histone coated strip wells were rehydrated and 5 µl of the desired dilutions of testing compounds and 7.5 µl of PARP enzyme (0.5 Unit/well) solution were added. This sat at room temperature for 10 minutes before the addition of 12.5 µl PARP cocktail. The wells were left at room temperature for 1 hour before washing toughly with PBS and PBS with 0.1% Triton X-100. After washing, 25 µl Strep-HRP was added to each well. Again, the wells sat at room temperature for 1 hour before repeating the washing process. After the wells were washed and dried, by patting on top of a paper towel, 50 µl of TACS-Sapphire was added and then wells were placed inside a drawer at room temperature for 15 minutes before adding 50 µl of 0.2 M HCl. The resulting absorbance at 450 nm was read with a Bio-rad benchmark Microplate reader (Bio-Rad, Hercules, CA) prior to GraphPad Prism 7 analysis.

### *In vivo* PARP activity inhibition

Exponentially growing V79 cells were treated with chemicals for 30 minutes before exposed to 2 mM hydrogen peroxide for 10 minutes. After hydrogen peroxide treatment, cells were washed with PBS and fixed in 4% paraformaldehyde for 15 minutes, followed by permeabilization in 0.2% Triton X-100 in PBS solution for 10 minutes. After blocking with 10% goat serum in PBS, anti-poly (ADP ribose) mouse monoclonal antibody (1:500 dilution in 10% goat serum) was added to the cells for incubation at 37 °C for 1 hour. Alexa 488 conjugated anti-mouse goat antibody (1:500 dilution in 10% goat serum) was used for the secondary antibody. After DNA staining with DAPI in SlowFade (Thermo Fisher Scientific, Waltham, MA), fluorescence images were obtained by Zeiss Axiophot fluorescence microscope equipped with Q-imaging Aqua Cooled CCD monochrome camera with Q-capture Pro software (Q-imaging, Surrey BC, Canada). Poly (ADP-ribose) formation observed as green fluorescence signals was quantified with Metamorph software. Green pixels per cell was scored for a minimum of 30 cells.

### Synthetic lethality DNA damage formation

Exponentially growing V79, V-C8, and gene corrected cells were exposed to 20 µg/ml of rosemary extract solution overnight. After treatment, cells were washed with PBS and fixed in 4% paraformaldehyde for 15 minutes followed by permeabilization in 0.2% Triton X-100 in PBS solution for 10 minutes. After blocking with 10% goat serum in PBS, Anti-gamma-H2AX mouse monoclonal antibody (1:300 dilution in 10% goat serum) was added to the cells for incubation at 37 °C for 1 hour. Alexa 488 conjugated anti-mouse goat antibody (1:500 dilution in 10% goat serum) was used for the secondary antibody. After DNA staining with DAPI in slowfade, 1 µm slices of fluorescence images were obtained by motorized Zeiss Axioskop fluorescence microscope equipped with CoolSNAP HQ2 Cooled CCD monochrome camera (Photometrics, Tucson, AZ) with Metamorph (Molecular Devices, Sunnyvale, CA) to obtain extended focus images. Massive DNA damage was categorized as more than 5 gamma-H2AX foci per cell.

### Statistics

A minimum of three independent experiments were carried out, consequent data was analyzed using GraphPad Prism 7 software. The data is presented as the mean ± standard error of the means. IC_50_ values (50% inhibitory concentration doses of the specific endpoints) were derived by fitting dose response curves using a sigmoidal dose response equation obtained by GraphPad Prism. Differences with a P < 0.05 were considered statistically significant, statistical comparison of mean values was performed using two-way ANOVA test with multiple comparisons.

### Data availability statement

All data generated or analyzed during this study are included in this published article.
